# Genetic Algorithm-Based Motion Estimation Method using Orientations and EMGs for Robot Controls

**DOI:** 10.3390/s18010183

**Published:** 2018-01-11

**Authors:** Jeongsook Chae, Yong Jin, Yunsick Sung, Kyungeun Cho

**Affiliations:** Department of Multimedia Engineering, Dongguk University-Seoul, Seoul 04620, Korea; jschae@dongguk.edu (J.C.); j.yong@dongguk.edu (Y.J.); sung@dongguk.edu (Y.S.)

**Keywords:** motion estimation, Bayesian probability, Myo device, orientation, EMG, genetic algorithm, weight

## Abstract

Demand for interactive wearable devices is rapidly increasing with the development of smart devices. To accurately utilize wearable devices for remote robot controls, limited data should be analyzed and utilized efficiently. For example, the motions by a wearable device, called Myo device, can be estimated by measuring its orientation, and calculating a Bayesian probability based on these orientation data. Given that Myo device can measure various types of data, the accuracy of its motion estimation can be increased by utilizing these additional types of data. This paper proposes a motion estimation method based on weighted Bayesian probability and concurrently measured data, orientations and electromyograms (EMG). The most probable motion among estimated is treated as a final estimated motion. Thus, recognition accuracy can be improved when compared to the traditional methods that employ only a single type of data. In our experiments, seven subjects perform five predefined motions. When orientation is measured by the traditional methods, the sum of the motion estimation errors is 37.3%; likewise, when only EMG data are used, the error in motion estimation by the proposed method was also 37.3%. The proposed combined method has an error of 25%. Therefore, the proposed method reduces motion estimation errors by 12%.

## 1. Introduction

Motion controller devices for user interfacing are utilized to recognize user’s motions or to control remote robots. Motion recognition is an approach that enables users to interact with a computer naturally by using wearable devices to capture their movements, positions, and gestures. These devices become increasingly smaller, more intelligent, and more varied in type and configuration. For example, motions are detected by measuring arm angles or muscle activities from a band worn on a arm [1], wearing a ring can allow for finger gestures to be utilized as input signals [2], and gloves can be sued to recognize users’ hand gestures [3]. Myo device, developed by Thalmic Labs, is a band-shaped gesture control device that is worn on one of arms [4]. 

Researchers have proposed motion estimation methods based on Bayesian probability to reduce the number of Myo device [5], but these methods have several problems. First, Bayesian probability lowers accuracy because it is calculated by dividing the Myo device data into sections without considering its distribution. Second, motion estimation accuracy is reduced when Bayesian probability is calculated without considering the associations of each of the x, y, and z orientations. Therefore, it is necessary to improve the Bayesian probability approach to motion estimation to increase estimation accuracy. In other words, accuracy can be increased by using additional data such as electromyogram (EMG) of Myo device.

This paper proposes a method for calculating Bayesian probabilities and estimating motions using EMG and orientation data from a Myo device. The final motions are determined by comparing the Bayesian probabilities of motions applying the weights that are calculated by the proposed method based on orientations and EMGs. In this paper, the measured raw data of subjects who are not calibrated for predefined motions are used. In this paper, only the orientation and EMG are utilized without using any velocity. Orientation data and EMG signals of a Myo device measured at a rate of 30 frames is utilized. 

The remainder of the paper is organized as follows: Section 2 introduces research on wearable devices. Section 3 proposes a method for Bayesian probability calculation and motion estimation based on multiple types of Myo device data. Section 4 describes the experimental method, results, and the analysis of those results. Finally, Section 5 outlines the conclusions and explores directions of future research.

## 2. Related Works

There are the diverse kinds of contactable wearable devices, such as Myo device and GestTrack3D Hand Tracker of GestTrackTek in USA [6]. Myo device is a built-in armband processor that recognizes motions by sensing the movements of the hands and arms delivered by the muscles through orientation, Inertial Measurement Units (IMU), and eight EMG sensors. Unlike Kinect and Leap Motion, which are widely known as motion recognition devices, Myo device and GestTrack3D Hand Tracker are Bluetooth-connected, so that they can be used remotely at up to 15 m.

When a Myo device is worn, the sensors measure muscle activity to determine the kind of gestures performed expressed as electrical signals. For example, an EMG extracts and amplifies the activity accompanying muscle contraction. The muscle sensor is composed of an amplifier, a high-pass filter (HPF), and a low-pass filter (LPF). EMG signals are amplified because the intensity of signals from muscle contractions is minute. The range of signals measured in the muscles is 10–2000 Hz, whereas the informative signals from the typical human’s muscles usually appears at 20–450 Hz. Therefore, signals of other frequencies can be regarded as noise. Myo device is equipped with an EMG, Bluetooth 4.0 communication, a three-axis accelerator, a three-axis gyroscope, and a three-axis magnetometer. Myo device can measure 25 finger and arm movements that are related to muscle activity, including movements more complex than simple hand gestures. It also measured the degrees of freedom (DOFs) of the hand, and can provide natural language interactions. Harrison et al. [1] studied the differences in recognition accuracy across user group in a system like Myo device, in which 10 sensors were attached to the arm sensor to learn the waveform using machine learning.

The Myo device also has a 9-axis Inertial Measurement Unit (IMU) that includes a 3-axis gyroscope, a 3-axis accelerometer, and a 3-axis magnetometer. The direction and movement of the wearer’s arm are measured by these units and determined by analyzing the spatial data provided. Orientation represents the positions of the band in terms of roll, pitch, and yaw. The angular velocity of Myo device is provided in vector form and the accelerometer represents the acceleration corresponding of Myo device. 

Strelow and Singh [7] proposed a motion estimation method using a camera and various sensors. Performance improved when the motion estimation method using the camera, the gyro sensor, and the acceleration sensor, as compared to when the camera was used independently. Gesture recognition through the integration of acceleration and EMG sensors [8] and the fast Fourier transform (FFT) of EMG sensors has been investigated when setting a specific EMG recognition interval [9]. Additional learning methods, such as a decision tree of acceleration sensor data and a k-nearest neighbor algorithm, have been studied for behavior recognition [10]. Further, behavior recognition has been performed using dynamic Bayesian networks of bio-signals and acceleration sensors [11].

A study of cube games using gesture recognition [6] recognized hand gestures through several EMG sensors and three-axis acceleration sensors. In addition, studies on gesture recognition have examined using EMG signals of static hand movements [12], and Wilcher motor control of wearable devices to recognize the EMG signals of neck muscles [13]. However, most of these studies are limited to a static motion, rather than a changing dynamic pattern of motion over time [8,13], or a motion that is dynamic, but clearly distinguishable [9]. On the other hand, there are also other research methods using genetic algorithm optimizations [14,15,16,17].

Since biological signals have very different body characteristics, it is necessary to use very complex algorithms, such as the classification of precise sensors and clear operation, learning, and processing using a large amount of data, and so the research scope is very limited. 

To improve motion estimation accuracy, the equipment used must also be improved, which can be costly. Kim et al. [5] proposed a motion estimation method using one Myo device sensor that estimates subordinate motion using data placed in dependency by the Bayesian probability of the data obtained from two Myo devices for motion estimation. To estimate motion, the sensor value section is determined after finding the interval based on all Myo device sensor value ranges, and the Bayesian probability is calculated when considering the number of sensor values measured in each section. However, most sensor values are contained in only a few intervals, and are excluded from others.

Lee [18] proposed an algorithm that redefines a data segment by redefining only the segment with the measured sensor value, and determines the motion to be meaningful data. However, there are limitations in solving this problem by considering only orientation data; we propose a motion estimation method based on weighted with Genetic algorithms and Bayesian probability, which suggests a Bayesian probability for orientation data and EMG data separately, and then selects the final motion by comparing the two in motion estimation. In this study, we define five basic operations and compute the motion probabilities that are estimated by various data characteristics to derive the final probability, enabling more accurate motion estimation under a variety of conditions. 

Recently, Myo’s EMG motion estimation study is Kim [19]. They suggested an algorithm to estimate the arm motion using the MYO armband attached to the upper arm. The motion of the lower arm is estimated through the EMG signal of the upper arm. The motion of the upper arm is estimated through the IMU sensor. They used an algorithm with sum, average, and rate based on the data measured on EMG of MYO. According to the results of this study, accurate motion estimation results can be confirmed at 0° and 90°. 

Nourhan T. [20] proposed a method to measure the muscle momentum according to the degree of muscle contraction during exercise using EMG data. The characteristics of the EMG obtained from the muscles are analyzed to classify the RMS (Root Mean Square) and the MDF (M-dimensional feature vector), and then the data set is learned using the artificial neural network (ANN). They are using ANN proposed two algorithms to predict muscle fatigue by classifying them as non-fatigue and fatigue.

Mendez, I. [21] proposed a method to solve the problem by decision tree using measured data from Myo’s EMG to determine user’s pose. After learning the measured data of 10 scissors, rocks, and beams, we randomly played the game and showed 78% of the results. Myo’s EMG signal is analyzed to show the related research on classification method for motion estimation as a research method that is applied to game after learning.

However, the Myo device is better suited to determine the relative position of the arm, not the absolute position. Myo device-related approaches can be applied to diverse fields, such as robot control and remote medical support. Since Myo device’s sensor data feature always provides a constant absolute coordinate system, the Myo device orientation starts measuring and is arranged in the current position with the movement of the next position and relative position coordinates.

In this sense, “relative” in this study is the Bayesian probability by taking Myo device on the upper arm and forearm of right arm in the learning stage and then taking into account their correlation. In the recognition phase, Myo device is put on the forearm of the arm, and then the Bayesian probability is determined and the motion is determined by selecting the motion having the Euclidian distance closest to the motion defined in the learning.

Therefore, in order to control remote or remote objects such as robot control, it is possible to perform more accurate and elaborate missions, by calculating the displacement of the relative coordinates from the current position starting point to provide various motion recognition information. The approach of wearing Myo devices on the upper arm and forearm is generalizable to other parts of the body, such as the legs.

In this paper, the methodology for motion estimation is as follows. We used the orientation and EMG raw data measured at 30 frame rates after wearing the upper arm and forearm of the right arm for the five defined stopping operations as input data. The data measured in two Myo devices are defined as independent motion and the Myo device worn at the upper arm is defined as the dependent motion. Learning is defined by defining the correlation in the learning stage. In the recognition phase, the final motion is determined by looking at the motion defined at the learning stage and the motion at the upper arm as the orientation and EMG raw data measured at one Myo device worn at the forearm of the arm for motion estimation. Detailed methodologies are described in detail in the following chapters.

## 3. The Myo Device Motion Estimation Framework

User’s motions can be estimated more accurately by utilizing multiple currently measured data. The following details the procedure of the proposed motion estimation method.

### 3.1. Measurement Methodology

**Figure 1:**
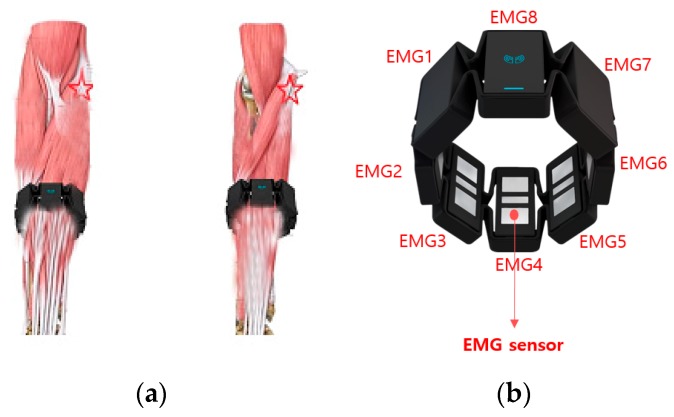
(**a**) Positioning of a Myo device for electromyogram (EMG) measurement, (**b**) eight EMG signals of one Myo device.

The proposed method utilizes a wearable Myo device. To estimate a user’s motions, Myo devices are set on the upper arm and forearm of one arm, and a subordinate relation of the two are set, for example, the upper arm can be defined as a dependency on the forearm. At the end of learning, the subordinate relationship enables motion estimation of the upper arm in subordinate relation to the independent motion of the forearm at the recognition phase. Figure 1 shows Myo devices attached to the forearm. In this paper, signal patterns are analyzed by measuring a user’s wrist movements with eight EMGs.

**Figure 2:**
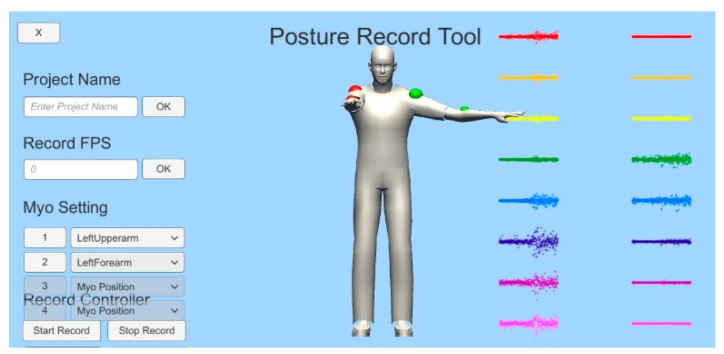
EMG signal recording.

**Figure 3:**
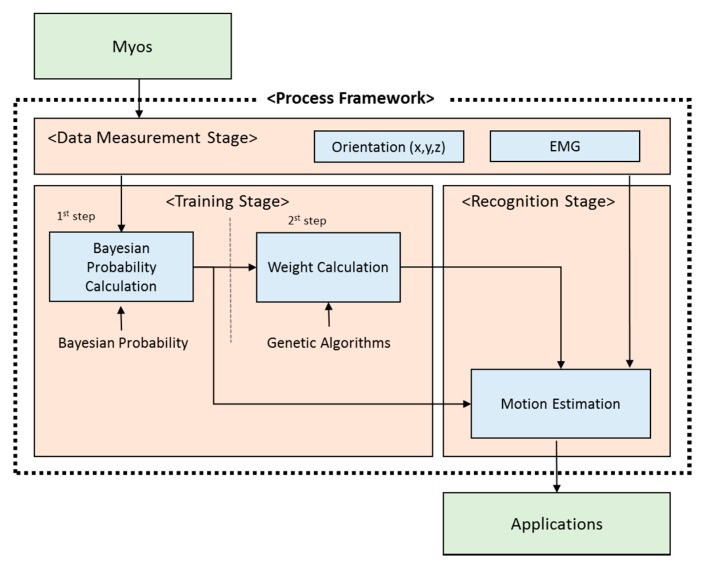
Components and structures of the proposed method.

Figure 2 shows the EMG signals acquired after wearing Myo devices on the upper arm and forearm. The eight EMGs have different signals depending on the muscle activity being measured. As shown in Figure 3b, one Myo device has eight EMG sensors built in the band. Therefore, the EMG signals corresponding to the corresponding muscles of the wearable area are input to each of the eight EMG channels. The human arm muscles are composed of several muscles with different strengths depending on the degree of development and the magnitude of the force, and all of the eight EMG signals show different measured values even when measured at the same time, according to the position of the EMG channel measured. However, when worn at the same position, a certain pattern is maintained depending on the motion.

The final motion is determined by selecting the motion corresponding to the signal with the highest Bayesian probability calculated by each signal.

The overall structure of the motion estimation approach is shown in Figure 3. First, Bayesian probability is obtained by using the orientation x, y, and z and EMG obtained from the Myo devices. Weights are calculated and then used to estimate the final motion.

The proposed method consists of data processing, training, and recognition stages. Orientation and EMG data are recorded with Myo devices. The orientation data are transferred to the Bayesian probability calculation step of the training stage. The calculated Bayesian probability is used in a genetic algorithm to obtain weights, which are then transferred to the motion estimation step in the recognition stage. 

### 3.2. Data Measurement

In the data processing phase, the Myo device measures data. Data that can be thus obtained are shown in Table 1. 

In the proposed method, only the orientation and EMG are used, but motion estimation accuracy can be improved using additional data of other types by applying the proposed method.

### 3.3. Traning Stage

**Figure 4:**
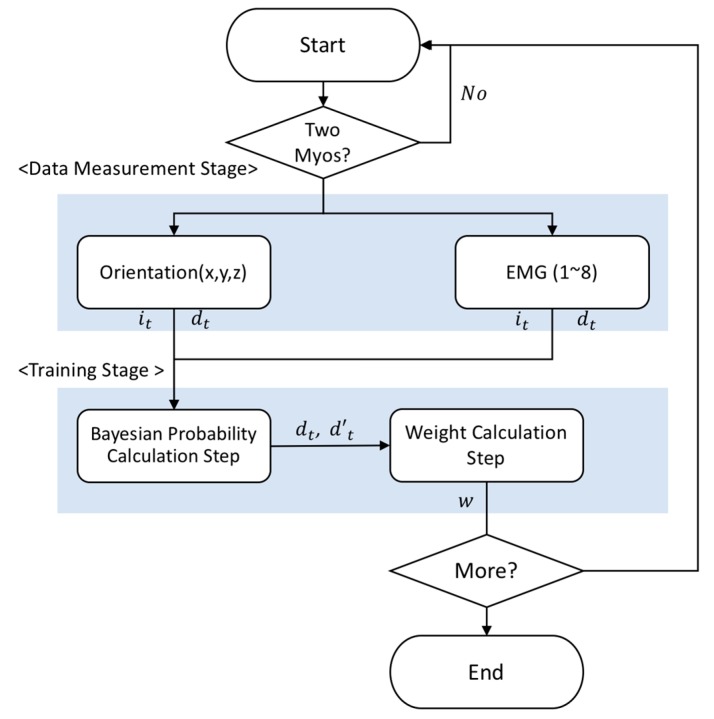
Training Stage.

Training consists of two steps. The orientation data and EMG input from the data processing phase are processed, as shown in Figure 4, in the Bayesian probability calculation.

The motions not worn by Myo device are estimated by the orientation and EMG of the motions worn by Myo device. Set the independent motion $i_{t}=[i^{x}{}_{t},\;i^{y}{}_{t}$,$\;i^{z}{}_{t}]$ and the dependent motion $d_{t}=\left[d^{x}{}_{t},\;d^{y}{}_{t},\;d^{z}{}_{t}\right].\;$Set $d\prime^{x}{}_{t}=\;\frac{argmax}{d^{x}{}_{i}}p(\lceil \frac{d^{x}{}_{i}}{\delta^{x}}\rceil |\lceil \frac{i^{x}{}_{t}}{\delta^{x}}\rceil ,\;\lceil \frac{\;i^{y}{}_{t}}{\delta^{y}}\rceil ,\;\lceil \frac{i^{z}{}_{t}}{\delta^{z}}\rceil )$, $d\prime^{y}{}_{t}=\;\frac{argmax}{d^{y}{}_{i}}p(\lceil \frac{d^{y}{}_{i}}{\delta^{y}}\rceil |\lceil \frac{i^{x}{}_{t}}{\delta^{x}}\rceil ,\;\lceil \frac{\;i^{y}{}_{t}}{\delta^{y}}\rceil ,\;\lceil \frac{i^{z}{}_{t}}{\delta^{z}}\rceil )$, and $d\prime^{z}{}_{t}=\;\frac{argmax}{d^{z}{}_{i}}p\left(\left\lceil \frac{d^{z}{}_{i}}{\delta^{z}}\rceil |\lceil \frac{i^{x}{}_{t}}{\delta^{x}}\rceil ,\;\lceil \frac{\;i^{y}{}_{t}}{\delta^{y}}\rceil ,\;\lceil \frac{i^{z}{}_{t}}{\delta^{z}}\right\rceil \right),$ where $d^{x}{}_{i},\;d^{y}{}_{i},d^{z}{}_{i}$ are the ith possible value of the values collected in advance, $\delta^{x}=\frac{\left(Max\left(i^{x}\right)-Min\left(i^{x}\right)\right)}{\zeta }$,$\;\delta^{y}=\frac{\left(Max\left(i^{y}\right)-Min\left(i^{y}\right)\right)}{\zeta },\;\delta^{z}=\frac{\left(Max\left(i^{z}\right)-Min\left(i^{z}\right)\right)}{\zeta }$ and ζ is the amount of sections.

**Figure 5:**
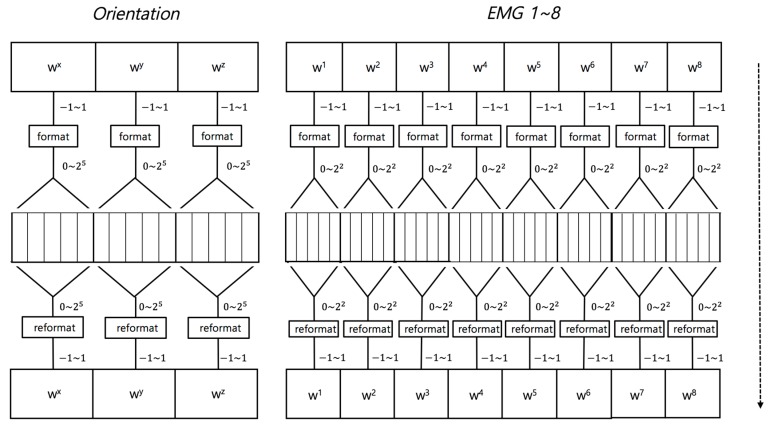
Data structure of the genetic algorithms.

The weight calculation step is shown in Figure 5. $f(w^{x}$,$w^{y},w^{z}$) is weight function. Each orientation is weighted and expressed as 15 bits for the genetic algorithm. The fitness function for weight calculation in the genetic algorithm is defined, as follows. The x, y, and z orientations are defined as fitness functions by defining the Euclidean distance as the closest value to the previously defined motion.

Each weight ranges from −1 to 1 in increments of 0.2. For orientation, three weights,$\;w^{x}$, $w^{y}$, and $w^{z}$, are utilized for $d^{x}{}_{t},\;d^{y}{}_{t}$,$\;\mathrm{and}\;d^{z}{}_{t}$, and are expressed as 15 bits for the genetic algorithm. The orientation fitness function is defined as shown in Equation (1).

(1) $$f\left(w^{x},\;w^{y},w^{z}\right)=\;\underset{i=1}{\sum }\sqrt{{\left(w^{x}\times d^{x}{}_{i}-d\prime^{x}{}_{t}\right)}^{2}+{\left(w^{y}\times d^{y}{}_{i}-d\prime^{y}{}_{t}\right)}^{2}+{\left(w^{z}\times d^{z}{}_{i}-d\prime^{z}{}_{t}\right)}^{2}\;}$$

where $w^{x}+w^{y}+w^{z}=1$. Therefore, each weight is normalized during the genetic algorithm process. For example, $w^{x}$, $w^{y}$, and $w^{z}$ are normalized by

(2) $$w^{x}=\frac{w^{x}}{w^{x}+w^{y}+w^{z}}\;,\;w^{x}=\frac{w^{y}}{w^{x}+w^{y}+w^{z}}\;,\;w^{x}=\;\frac{w^{z}}{w^{x}+w^{y}+w^{z}}\;$$

The dependent motion $d^{\prime }{}_{t}=\left[d^{x}{}_{t},\;d^{y}{}_{t},\;d^{z}{}_{t}\right]$ are also calculated by EMGs, $i^{1}{}_{t},\;i^{2}{}_{t}$, …,$\;i^{8}{}_{t}$ and $d^{1}{}_{t},\;d^{2}{}_{t}$, …,$\;d^{8}{}_{t}$, obtaining the weights, $w^{1}+w^{2}+\cdots +w^{8}=1,$ for EMGs by the Generic algorithm.

### 3.4. Recognition Stage

**Figure 6:**
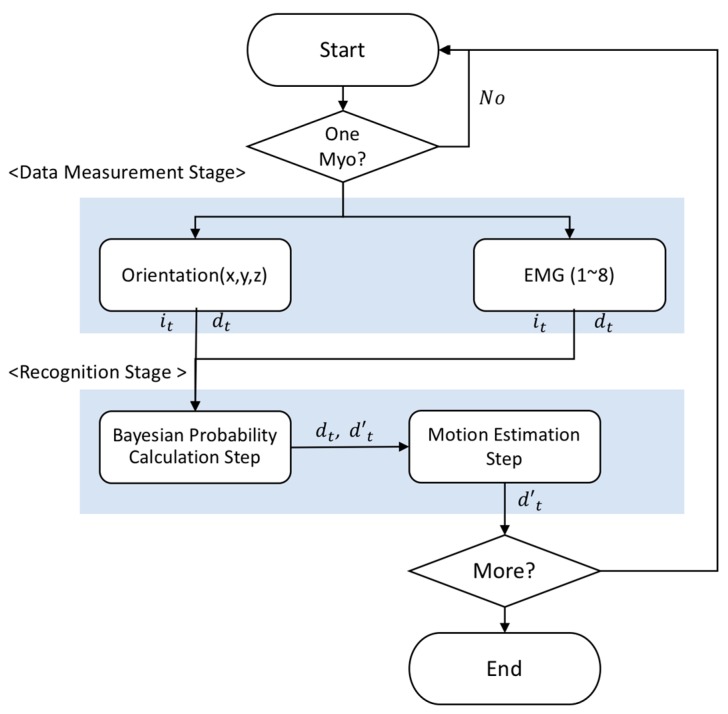
Recognition stage.

The Bayesian probability calculation from the orientation and EMG data in the motion estimation step reflects the weights determined by the genetic algorithm as shown in Figure 6. 

Then, the Bayesian probability updated by the weights is calculated, and final motion is estimated. Of the Bayesian probabilities calculated from the x, y, and z orientations and EMG data, the motion with the highest probability as the final motion is selected as in Equation (3). Handling multiple data types can more accurately estimate motions than traditional methods.

(3) $$p^{o}{}_{t}=\;\frac{p(\lceil \frac{d^{x}{}_{i}}{\delta^{x}}\rceil |\lceil \frac{i^{x}{}_{t}}{\delta^{x}}\rceil ,\;\lceil \frac{\;i^{y}{}_{t}}{\delta^{y}}\rceil ,\;\lceil \frac{i^{z}{}_{t}}{\delta^{z}}\rceil )+p(\lceil \frac{d^{y}{}_{i}}{\delta^{y}}\rceil |\lceil \frac{i^{x}{}_{t}}{\delta^{x}}\rceil ,\;\lceil \frac{\;i^{y}{}_{t}}{\delta^{y}}\rceil ,\;\lceil \frac{i^{z}{}_{t}}{\delta^{z}}\rceil )+p\left(\left\lceil \frac{d^{z}{}_{i}}{\delta^{z}}\rceil |\lceil \frac{i^{x}{}_{t}}{\delta^{x}}\rceil ,\;\lceil \frac{\;i^{y}{}_{t}}{\delta^{y}}\rceil ,\;\lceil \frac{i^{z}{}_{t}}{\delta^{z}}\right\rceil \right)}{3}$$

(4) $$p^{e}{}_{t}=\;\frac{p\left(\left\lceil \frac{d^{1}{}_{i}}{\delta^{1}}\rceil |\lceil \frac{i^{1}{}_{t}}{\delta^{1}}\rceil ,\;\lceil \frac{i^{2}{}_{t}}{\delta^{2}}\rceil ,\;\ldots ,\;\lceil \frac{i^{8}{}_{t}}{\delta^{8}}\right\rceil \right)+\ldots +p\left(\left\lceil \frac{d^{8}{}_{i}}{\delta^{8}}\rceil |\lceil \frac{i^{1}{}_{t}}{\delta^{1}}\rceil ,\;\lceil \frac{i^{2}{}_{t}}{\delta^{2}}\rceil ,\ldots ,\;\lceil \frac{i^{8}{}_{t}}{\delta^{8}}\right\rceil \right)}{8}$$

(5) $$d^{\prime }{}_{t}=\;\{\begin{array}{c} if\;p^{o}{}_{t}>\;p^{e}{}_{t}\\ d^{\prime }{}_{t}\;by\;i^{x}{}_{t},i^{y}{}_{t},i^{z}{}_{t}\\ else\;d^{\prime }{}_{t}\;by\;i^{1}{}_{t},i^{2}{}_{t},i^{3}{}_{t},i^{4}{}_{t},i^{5}{}_{t},i^{6}{}_{t},i^{7}{}_{t},i^{8}{}_{t} \end{array}$$

## 4. Experiments and Analysis

### 4.1. Overview of Experimental Environments

**Figure 7:**
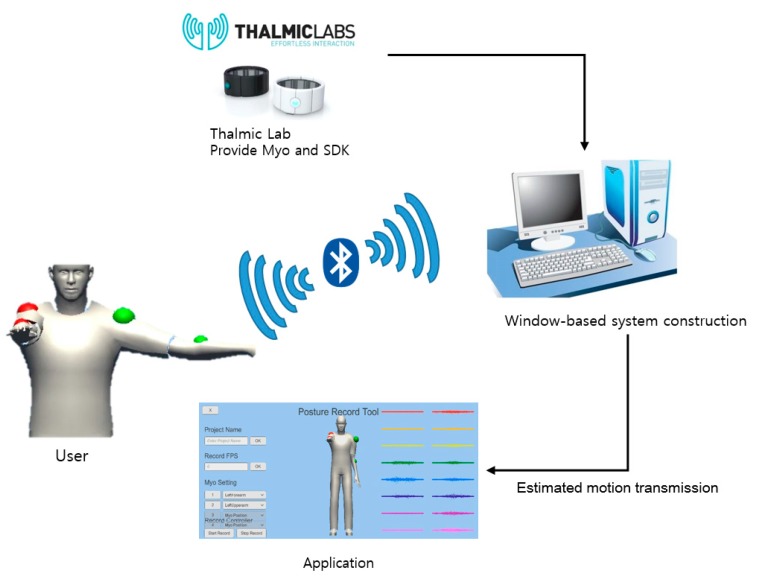
System of Myo device Motion Estimation.

The experimental environment of this study was implemented with Unity 5.4.2 based on the Win10 operating system and four Myo device devices based on Bluetooth 2.0 were used. Figure 7 shows the system conceptual diagram for constructing the experimental environment. We implemented the proposed algorithm and compared it with the traditional algorithm in an experiment for which we defined five user motions.

First, the subject calibrates his motions by the developed application connecting with Thalmic Labs’ Myo Connect manager. When the subject moves a lot, he needs to calibrate again from the beginning. In addition, the calibration is deducted by performing four pre-defined gestures, finger spread, wave-in, wave-out, and relaxed state gestures, after wearing a Myo device.

### 4.2. Motion Definitions

**Figure 8:**
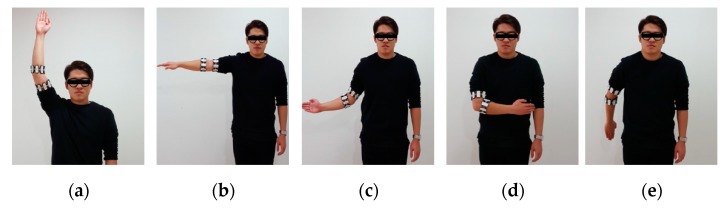
This is a figure, the five defined postures. (**a**) Screen shoots of Flexion posture; (**b**) Screen shoots of Extension posture; (**c**) Screen shoots of Abduction posture; (**d**) Screen shoots of Internal posture; and, (**e**) Screen shoots of External posture.

The subjects’ motions were defined by five representative major motions of the arm. To learn these motions, Myo devices were worn on the upper and lower parts of the subjects’ left arm. Figure 8 defines these motions showing the right arm, but they are defined in the same way for the left arm. Flexion refers to the downward motion of the lower arm, the extension of the lower arm. It is a motion that is 90° to the body, rising from the side under the Abduction. Internal is the motion in which the arms are folded inward from the center of the waist. External is a motion that stretches 180° outward from the center-right navel position, as opposed to internal motion. In the learning stage, five subjects performed the predefined movements 10 times with an interval of 10 min after wearing Myo devices on the upper arm and forearm.

The learned motion is recorded for each user and stored in the user table, and is used to estimate the most accurate motion in the recognition stage. In addition, motion recognition features are recorded for each user, and user-independent motion estimation could be possible using the average value obtained in the experimental data.

### 4.3. Result of the Proposed Method by Orientation

**Figure 9:**
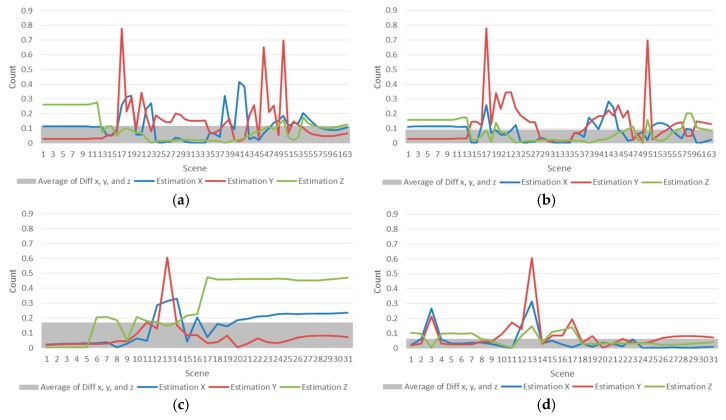

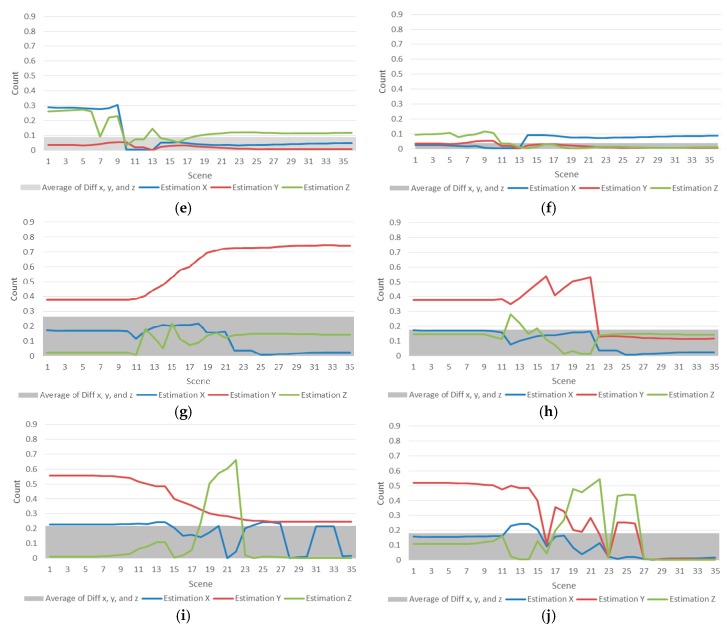
Differences in orientation-based estimation between the traditional method [17] and the proposed method, and the average of cumulative errors in the x, y, and z orientations. (**a**,**c**,**e**,**g**) and (**i**) compare the differences in the estimated motion of the x, y, and z orientations using the previous research method. (**b**,**d**,**f**,**h**,**j**) showing the differences in the estimation results of these orientations when they are weighted by the proposed algorithm.

Figure 9 shows the result of the orientation algorithm experiment, and compares the motion estimation results of the proposed method and the traditional research [17]. Figure 9a,b shows the experimental results of Abduction for orientation and (c,d) are experimental comparison results of Flexion motions. The other figures (e,f) are experimental comparison results of Extension motions, (g,h) are experimental results of External motions, (i,j) are results of Internal motions.

The histogram behind the graph represents the mean value of the total error of the motion estimation. The histogram of the sum of the errors shows that the motion estimation error of the proposed algorithm is small. (f,h,j) show smaller differences than (e,g,i).

As shown in Table 2, the sum of the total errors for the motion estimation result of the previous and newly proposed methods is 257.0418. The total sum of the errors of the motion estimation of the previous method was 95.88, which was an error of 37.3%; the error rate of the proposed method is 65.1, which is an error rate of 25%. The sum of the differences of the motion estimation results from EMG data was 96.03, indicating that its error rate was also 37.3%. With this comparison, we can see that the proposed method reduced estimation errors from orientation data by 12% as compared with the motion estimation result of the previous study.

**Figure 10:**
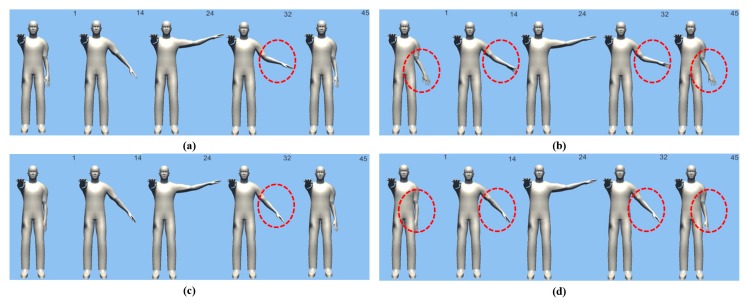
The following figure is a comparison of the experimental results of the previous studies on the Abduction motion and the proposed studies. Motion Estimation result Screen of Proposed Method (**a**) Screenshot of raw orientation x, y, and z (**b**) Shows the results of the previous research (**c**) Shows the motion estimation result after applying the weight of the genetic algorithm (**d**) Shows the Bayesian probability estimation result that reflects the weight.

**Figure 11:**
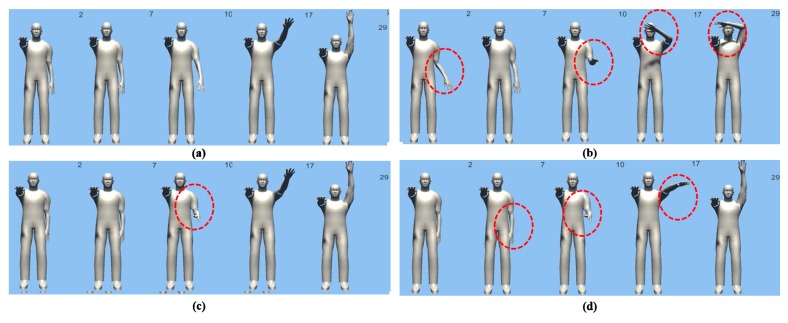
The following figure is a comparison of the experimental results of the previous studies on the Internal motion and the proposed studies. Motion Estimation result Screen of Proposed Method (**a**) Screenshot of raw orientation x, y, and z (**b**) Shows the results of the previous research (**c**) Shows the motion estimation result after applying the weight of the genetic algorithm (**d**) Shows the Bayesian probability estimation result that reflects the weight.

Figure 10 and Figure 11 show the results of motion estimation based on the original orientation-based method of and the proposed weight-based Bayesian probability method using an avatar. 

The algorithm comparisons for the main frames in all estimated motions are shown in red and blue circles. As can be seen from the original motions, the proposed algorithm showed improved results.

### 4.4. Result of the Proposed Method by EMG Data

**Figure 12:**
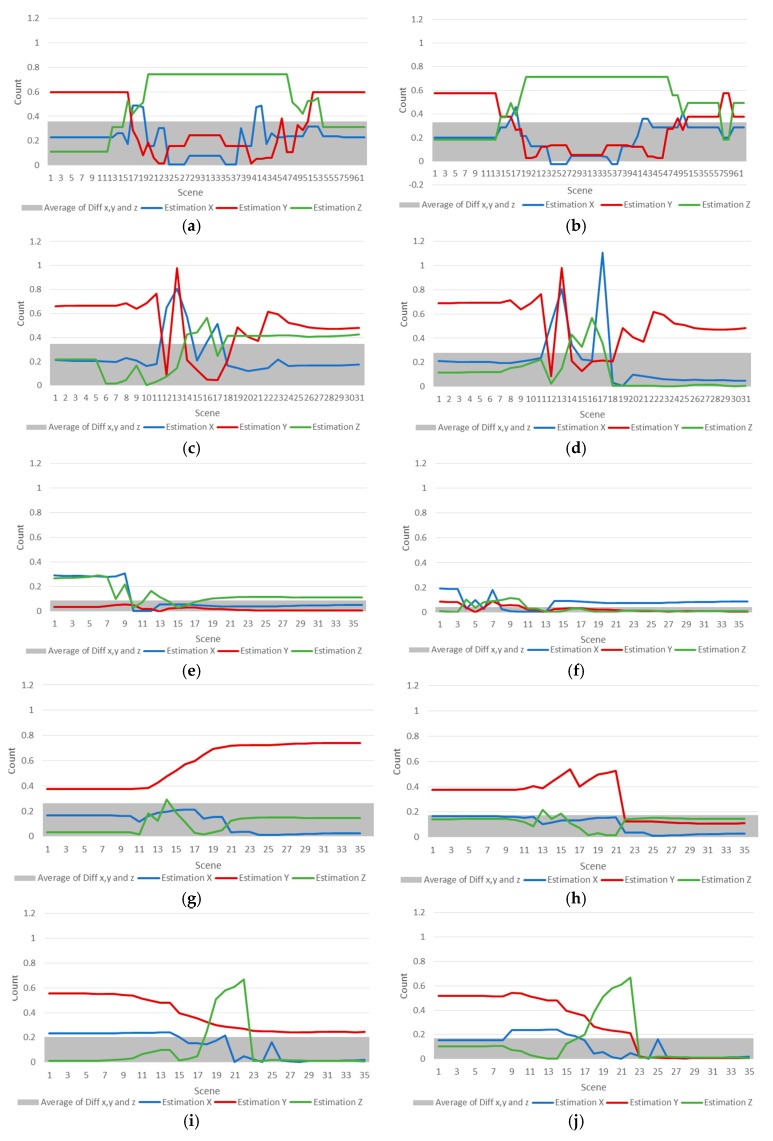
Differences in EMG estimation between the traditional method [17] and the proposed method, and the average of the cumulative x, y, and z error. (**a**,**c**,**e**,**g**,**i**) compare the differences in the estimated motion of the x, y, and z EMG using the previous research method. (**b**,**d**,**f**,**h**,**j**) show the EMG based motion estimation results using Bayesian probability that reflects the weights calculated by the genetic algorithm.

The results of the algorithm experiment on EMG data are shown in Figure 12, which graphs the differences between the previous study and the raw orientation of the proposed method. 

Figure 12 shows the experimental result of Abduction for EMGs, (a,b) are graphs of experimental results of Abduction motions, and (c,d) are comparison experimental results of Flexion motions. The other figures (e,f) are comparison experimental results of Extension motions, (g,h) are experimental results of External motions, (i,j) are results of Internal motions. Figure 12a,c,e,g,i compares the differences in the estimated motion of the x, y, and z EMG, using the previous research method. Figure 12b,d,f,h,j shows the EMG based motion estimation results using Bayesian probability, which reflects the weights calculated by the genetic algorithm. The graphs show the errors produced with the original data. The closer the orientation was to 0, the lower the estimation error was. The background histogram shows the average of the total errors. The graph also shows that the average errors of the newly proposed algorithm is also lower. Figure 12f,h,j shows a smaller difference when compared to Figure 12e,g,i.

## 5. Conclusions

In this paper, we proposed a Bayesian probability-based motion estimation algorithm that reflects the weight of Myo device signals by utilizing a genetic algorithm. In the training phase, we showed that upper-arm motions can be estimated by learning Bayesian probability with only one lower-arm Myo device after learning using two Myo devices: one on each of the upper and lower part of one arm. In addition, the performance improved when recalculating the Bayesian probability to reflect the weights calculated by the genetic algorithm. Experimental results showed that the data used for motion estimation are complementary by using two types of data: orientation and EMG.

The proposed algorithm, using a composite of orientation and EMG data, reduced motion estimation errors by 12% as compared with previous methods. Motion estimation errors from EMG data were also 37%, which agrees with traditional studies. In the future, it is necessary to study robot controls by transferring estimated users’ motions using a Myo, as proposed in this paper.

## Figures and Tables

**Table 1:** Myo device dataset format [14].

Myo device Data Structure	Orientation (x, y, z)
	Acceleration
	EMGs (1–8)
	Gyroscope
	IMU (Pitch, Roll, Yaw)

**Table 2:** Difference in the total sum of the difference of the original and estimated orientations.

	Bayesian Probability			Genetic Algorithms			EMG		
	X	Y	Z	X	Y	Z	X	Y	Z
Flexion	4.518535	2.38598	8.767333	1.708836	2.743719	2.108969	2.659626	2.718015	5.602758
Extension	3.767039	0.783646	5.044406	2.817137	0.937607	0.9398	5.415701	0.775235	4.554835
Abduction	7.152997	8.290485	6.407354	5.025141	8.030151	4.713921	7.646425	9.640942	5.725771
Internal	4.699618	13.25015	3.369718	3.674614	9.598146	4.42801	4.019890	10.22330	7.955438
External	3.736156	20.15425	3.554185	3.522207	10.38686	4.48866	4.483504	20.19237	4.422342
Total	23.87435	44.86451	27.143	16.74793	31.69649	16.67936	24.22515	43.54986	28.26114
	95.88 (37.3%)			65.1 (25%)			96.03 (37.3%)		
	257.0418								
